# Mast Cells as Important Regulators in Autoimmunity and Cancer Development

**DOI:** 10.3389/fcell.2021.752350

**Published:** 2021-10-12

**Authors:** Christine N. Noto, Stella G. Hoft, Richard J. DiPaolo

**Affiliations:** Department of Molecular Microbiology and Immunology, Saint Louis University School of Medicine, Saint Louis University, St. Louis, MO, United States

**Keywords:** mast cell, autoimmunity, cancer, rheumatoid arthritis, multiple sclerosis, type 1 diabetes

## Abstract

Mast cells are an essential part of the immune system and are best known as important modulators of allergic and anaphylactic immune responses. Upon activation, mast cells release a multitude of inflammatory mediators with various effector functions that can be both protective and damage-inducing. Mast cells can have an anti-inflammatory or pro-inflammatory immunological effect and play important roles in regulating autoimmune diseases including rheumatoid arthritis, type 1 diabetes, and multiple sclerosis. Importantly, chronic inflammation and autoimmunity are linked to the development of specific cancers including pancreatic cancer, prostate cancer, colorectal cancer, and gastric cancer. Inflammatory mediators released from activated mast cells regulate immune responses and promote vascular permeability and the recruitment of immune cells to the site of inflammation. Mast cells are present in increased numbers in tissues affected by autoimmune diseases as well as in tumor microenvironments where they co-localize with T regulatory cells and T effector cells. Mast cells can regulate immune responses by expressing immune checkpoint molecules on their surface, releasing anti-inflammatory cytokines, and promoting vascularization of solid tumor sites. As a result of these immune modulating activities, mast cells have disease-modifying roles in specific autoimmune diseases and cancers. Therefore, determining how to regulate the activities of mast cells in different inflammatory and tumor microenvironments may be critical to discovering potential therapeutic targets to treat autoimmune diseases and cancer.

## Introduction

Mast cells were discovered by Paul Ehrlich in 1878 but are estimated to have first appeared approximately 500 million years ago (Crivellato et al., 2003). They are present in all classes of vertebrates and are important modulators of allergic and anaphylactic inflammatory responses (Frossi et al., 2018). Mast cells also aid in tissue repair, wound healing, and the formation of new blood vessels (angiogenesis), as well as innate and adaptive immune responses. However, mast cells and their inflammatory mediators can be both protective and damage-inducing. Increased mast cell density and activation are associated with multiple autoimmune diseases including rheumatoid arthritis (RA), type 1 diabetes (T1D), and multiple sclerosis (MS; Walker et al., 2012). Importantly, these autoimmune diseases are linked to increases in cancer incidence in affected individuals (Mellemkjaer et al., 1996; Vigneri et al., 2009; Grytten et al., 2020). Additionally, mast cells are present in increased numbers within the tumor microenvironments of colorectal, gastric, and pancreatic cancers (Secor et al., 2000; Lee et al., 2002; Ribatti et al., 2010; Dalton and Noelle, 2012; Varricchi et al., 2017). Chronic inflammation and autoimmunity are linked to cancer development and mast cells may play an important part in facilitating carcinogenesis through their ability to regulate inflammatory responses.

Derived from CD34^+^ hematopoietic stem cells in the bone marrow, mast cells circulate the body in an immature form as mast cell progenitors and mature in target tissues (Moon et al., 2010). Migration and localization of mast cell progenitors to target tissues is mediated by chemokines and interactions between surface adhesion molecules. For example, skin mast cell progenitors adhere to fibroblasts via α4β7 integrin. Upon contact with these fibroblasts, mast cells downregulate the homing chemokine receptors CXCR2 and CX3CR1, hindering further migration (Leist et al., 2017; Frossi et al., 2018). CXCR2 expression on mast cells directs migration to the small intestine and localization is mediated by β7 integrin, α4β7 integrin, vascular cell adhesion molecule 1, and mucosal addressin cell adhesion molecule 1 (Gurish et al., 2001; Abonia et al., 2005). Mast cells are widely dispersed throughout the body, they are primarily found in areas with direct environmental contact (e.g., skin, mucosal tissues) but can also be found associated with blood and lymphatic vessels (Walker et al., 2012).

The growth factors and cytokines present in the surrounding microenvironment of the target tissue influence mast cell phenotype and function giving mast cells a high degree of heterogeneity (Gri et al., 2012; da Silva et al., 2014). Mouse and human mast cells have been historically categorized into two polarized subtypes based on their tissue distribution and content of their cytoplasmic storage granules. Subtypes of human mast cells are defined by the production of proteases (tryptase and/or chymase) while murine mast cells are classified based on location (mucosal or connective) (Elieh Ali Komi et al., 2020). Murine mucosal mast cells are differentiated via the expression of mouse mast cell protease (MCPT)-1 and -2 whereas connective tissue mast cells express MCPT-4, -5, -6, and -7 (Moon et al., 2010). Murine mast cells have been shown to transdifferentiate between phenotypes as a result of changing microenvironmental conditions (Kitamura, 1989). Similar heterogeneity is also observed in human mast cell populations. Human mast cells are classified as tryptase-only (MC_*T*_), chymase-only (MC_*C*_), or containing both tryptase and chymase (MC_*TC*_; Irani and Schwartz, 1994; Welle, 1997). Mast cell distribution in human tissues often contain mixed populations of mast cells (Welle, 1997). Transcriptional analysis of mast cells isolated from the skin, tongue, esophagus, trachea, and peritoneal cavity of mice determined that mast cells are one of the most transcriptionally diverse cell types of the immune system and display tissue-specific regulation of their transcriptomes (Dwyer et al., 2016). Secreted factors within the tissue regulate mast cell activities and functions creating a diverse population of mast cells (Wernersson and Pejler, 2014). The heterogeneity of mast cells in tissues is much more complex than the protease- or location-based historical classification and is likely ever changing in accordance with the dynamic microenvironment of the tissue. Differences between mast cell phenotypes include variances in granule contents, cytokine production, and surface receptor expression. The everchanging microenvironment results in a diverse population of mast cells within a single target tissue making mast cell heterogeneity a complex and robust topic that is discussed in depth in other reviews (Irani and Schwartz, 1994; Welle, 1997; Moon et al., 2010; Frossi et al., 2018).

Upon activation, mast cells can degranulate and release inflammatory mediators associated with allergy and anaphylaxis as well as aid in the protection against invading pathogens (Krystel-Whittemore et al., 2015; Elieh Ali Komi et al., 2020). These mediators can be either pre-formed or *de novo*, also referred to as “neosynthesized mediators.” Pre-formed mediators (including histamine, proteases, serotonin, heparin, and TNF among others) are stored in the cytoplasmic granules and released upon degranulation. Histamine, an abundant pre-formed mediator, promotes vasodilation and increased capillary permeability that aids in the recruitment of immune cells to the site of inflammation (Riley and West, 1952). Prostaglandins, leukotrienes, and various cytokines and chemokines make up a portion of neosynthesized mediators that are produced and secreted following mast cell activation in response to environmental stimuli (Walker et al., 2012; Elieh Ali Komi et al., 2020). Specific cytokine neosynthesized mediators include anti-inflammatory TGFβ, IL-10, and the proinflammatory T-helper (Th) 1 and Th2-associated cytokines IL-4, IL-6, and IFNγ (da Silva et al., 2014). The inflammatory molecules in the granules vary between mast cell subtypes, which are heterogeneous within a given tissue. Therefore, a single activation signal has the potential to activate different mast cell subtypes resulting in the release of an assortment of molecules with different effector functions (Frossi et al., 2018). Early release of preformed mediators like histamine, prostaglandin D2 (PGD2), chemokine ligand 2 (CCL2), vascular endothelial growth factor (VEGF)-A, and TNF during an inflammatory response aids in increased epithelial cell permeability and vasodilation and promotes the recruitment of other effector cells (Moon et al., 2010). Mast cells communicate with the adaptive immune system by priming T cells through antigen presentation via major histocompatibility complex (MHC)-I or MHC-II and influencing dendritic cell migration and function via the release of IL-1β and TNF (Malaviya et al., 1996; Poncet et al., 1999; Suto et al., 2006; Galli et al., 2008; Reuter et al., 2010; Walker et al., 2012; Table 1).

**TABLE 1:** Mediators and activities.

Mediator(s)	Activities	References
Histamine	Promotes vascular permeability, vasodilation, angiogenesis, inhibition of Th1 and Th2 responses	Riley and West, 1952
PGD2, CCL2, VEGF-A, TNF	Promote epithelial cell permeability, vasodilation, recruitment of effector cells	Moon et al., 2010
IL-1β, TNF	Influence dendritic cell migration and function	Malaviya et al., 1996; Poncet et al., 1999; Suto et al., 2006; Galli et al., 2008; Reuter et al., 2010; Walker et al., 2012
TGFβ, IL-6, IL-23	Differentiation of CD4^+^ T cells into Th17	Piconese et al., 2009; Zhu and Paul, 2010
TGFβ + IL-2	Differentiation of CD4^+^ T cells into Tregs	Piconese et al., 2009; Zhu and Paul, 2010
IL-6	Inhibition of Treg differentiation and favors Th17 differentiation	Piconese et al., 2009; Zhu and Paul, 2010; Solt and Burris, 2015; Betto et al., 2017
IL-4	Differentiation of CD4^+^ T cells into Th2	Malbec and Daëron, 2007
IL-17, IL-13, GM-CSF, MCP-1, MIP-1a	Immune cell recruitment, released by IL-33-mediated mast cell activation	Robbie-Ryan and Brown, 2002; Hueber et al., 2010; Xu et al., 2010
CCL5, SCF	Mast cell chemoattractants	Lock et al., 2002; Couturier et al., 2008
SCF, VEGF, CXCL8/IL-8	Attracts mast cells to TME	Varricchi et al., 2017
CXCL12	Attracts mast cells to TME and promotes release of pro-angiogenic mediators by mast cells	Jorpes, 1959; Theoharides et al., 1982; Kim et al., 1993; Kryczek et al., 2005; Beer et al., 2008; Carmeliet and Jain, 2011; Kolset and Pejler, 2011; Vizio et al., 2013; Derakhshani et al., 2019
IL-10, TGFβ	Suppression of immune response	Riley and West, 1952; Nakae et al., 2005; Melillo et al., 2010; Ribatti et al., 2010; da Silva et al., 2014; Krystel-Whittemore et al., 2015; Derakhshani et al., 2019
Adenosine	Suppression of immune response and promotes histamine secretion from mast cells	Riley and West, 1952; Marquardt et al., 1984; Gottfried et al., 2012; Piliponsky et al., 2012
CXCL8/IL-8	Induction of epithelial-to-mesenchymal transition	Scheel and Weinberg, 2012; Visciano et al., 2015; Zheng et al., 2015
Heparin, VEGF-A	Angiogenesis	Jorpes, 1959; Theoharides et al., 1982; Kim et al., 1993; Kryczek et al., 2005; Beer et al., 2008; Carmeliet and Jain, 2011; Kolset and Pejler, 2011; Vizio et al., 2013; Derakhshani et al., 2019
IL-33	Mast cell activation	Theoharides et al., 2010; Byrne et al., 2011; Chan et al., 2012; Rivellese et al., 2015; He et al., 2018

**FIGURE 1:**
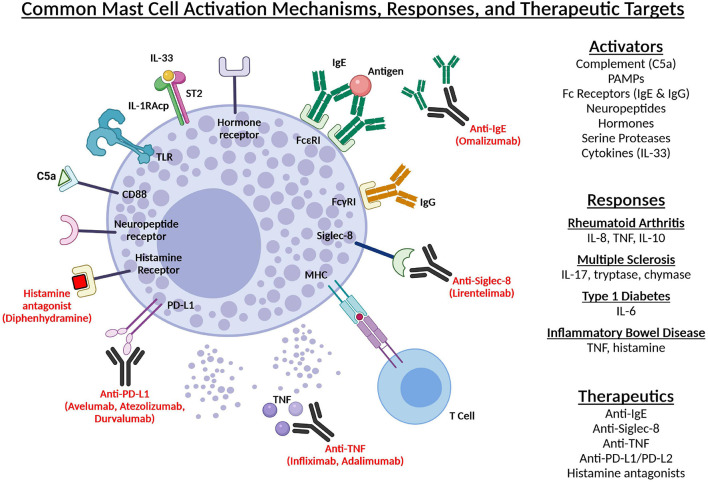
Common mast cell activation mechanisms, responses, and therapeutic targets. A summary of various methods of mast cell activation, mast cell responses, their associated autoimmune diseases, and therapeutic targets. Mast cells can be activated via toll-like receptors, complement anaphylatoxins, Fc receptors (FcεR and FcγR), neuropeptides, hormones, and cytokines. In individuals with rheumatoid arthritis, IL-33-mediated mast cell activation increases IL-8, TNF, and IL-10 production. Increased IL-17, tryptase, and chymase have been observed in the brains of patients with multiple sclerosis. IL-6 released by activated mast cells in the pancreas favors T cell differentiation into a Thl7 phenotype associated with type 1 diabetes. TNF-positive mast cells and increased histamine and histamine metabolites are observed in patients with inflammatory bowel disease. Various therapeutics and their mechanism of action are shown in red. Created with Biorender.com.

Mast cells are one of the first immune cells to interact with foreign antigen or pathogen. They can be activated via toll-like receptors, complement anaphylatoxins, pathogen-associated molecular patterns (PAMP), Fc receptors (FcεR and FcγR), neuropeptides, hormones, serine proteases, immunoglobulin light chains, and cytokines (Alvarez-Errico et al., 2009; Moon et al., 2010; Min et al., 2020). Monomeric IgE bound to FcεRI promotes survival and priming of mast cells. Cross-linking of IgE- or IgG-antigen complexes induces mast cell degranulation (Kawakami et al., 2005). Additionally, mast cells and T cells exist in close proximity in inflamed tissue and direct contact of mast cells with activated T cells can induce mast cell activation (Baram et al., 2001; Figure 1). It was also recently shown that mast cells can be activated by tumor- and T cell-derived microvesicles (Shefler et al., 2014; Salamon et al., 2020). Mast cells regulate the inflammatory response through direct cell-cell contact with neighboring immune cells or via the release of mediators. T regulatory cells (Tregs), T effector cells, and mast cells often co-localize at sites of inflammation, T cell priming, and specific tumor microenvironments. Tregs are important components of the immune system playing the crucial role of regulating immune responses. Tregs constitutively express OX40 and its ligand (OX40L) is constitutively expressed on mast cells. Via the OX40-OX40L axis, Tregs can downregulate FcεRI expression on mast cells and inhibit FcεRI-mediated mast cell degranulation. Conversely, activated mast cells can reduce T effector cell susceptibility to Treg suppression via the release of IL-6 and OX40 engagement (Gri et al., 2008; Kashyap et al., 2008; Piconese et al., 2009). Differentiation of naïve CD4^+^ T cells into Th17 cells can be accomplished during T cell activation through signaling with a combination of TGFβ, IL-6, IL-23. TGFβ along with IL-2 results in differentiation into Tregs, however, this can be inhibited by IL-6. Mast cells produce TGFβ, IL-23, and IL-6 and can therefore influence the frequency of Tregs and Th17 cells at co-localization sites (Piconese et al., 2009; Zhu and Paul, 2010). This is important to note as IL-17-producing Th17 cells are associated with disease development in mouse models of multiple sclerosis, rheumatoid arthritis, and type 1 diabetes (Hemdan et al., 2010). Mast cells also release IL-4 which polarizes T cells to a Th2 phenotype further highlighting the regulatory and/or modulatory roles of T cell-mast cell interactions that affect various aspects of the immune response (Malbec and Daëron, 2007; Table 1).

Multiple different mast cell deficient mouse models have been developed to study various aspects of mast cell function. Stem cell factor (SCF), known as c-Kit ligand, is important for mast cell maturation, proliferation, and survival (Okayama and Kawakami, 2006). SCF-induced c-Kit dimerization is required for mast cell development (Gilfillan et al., 2011; Amagai et al., 2015). C-Kit is expressed by hematopoietic stem cells, melanocytes, and germ cells and is mapped to the white spotting locus (W) (Orfao et al., 2007). Two mast cell-deficient mouse models have been developed using mutations in SCF or c-Kit, WBB6F1-Kit^*W/W–v*^ and C57BL/6-Kit^*W–sh*^. Kit^*W*^ is a point mutation preventing expression of Kit on the cell surface. Kit^*W–v*^ is a mutation in the c-Kit tyrosine kinase domain that reduces kinase activity of the receptor. Kit^*W–sh*^ is an inversion mutation that affects regulatory elements upstream of the c-Kit transcription start site. These mutations inhibit mast cell development; however, these genes are not specific to mast cells and affect other lineages including stem cells, neutrophils, and germ cells (da Silva et al., 2014). Due to off-target effects, Kit-independent mast cell-deficient mice have been developed including a mouse line that utilizes the expression of Cre recombinase from mast cell carboxypeptidase A3 (CPA3). *Cpa3* locus driven Cre recombinase expression selectively inhibits mast cell development. Besides a small reduction in basophils, these mice show very few off-target effects (Feyerabend et al., 2011). Another example of a Kit-independent model is the Mcpt5-Cre; R-DTA mice. These mice utilize Cre-specific depletion of mast cells in which the diphtheria toxin alpha chain is produced only in Cre-expressing cells (Reber et al., 2012). These mouse models have been used in countless studies to elucidate how mast cells contribute to the development and progression of various cancers and autoimmune diseases.

Activated mast cells have been found to have a pathophysiological role in inflammatory conditions of the skin, airways, and intestine [inflammatory bowel disease (IBD), Crohn’s disease, and ulcerative colitis] (Moon et al., 2010). Studies have found an increase in mast cell density and activation in many T cell-mediated diseases including RA, T1D, and MS. (Mekori, 2004; Maruotti et al., 2007; Sayed et al., 2008; Walker et al., 2012). The purpose of this review is to summarize the role(s) of mast cells in autoimmune disease development and their role in facilitating cancer development.

## Mast Cells in Autoimmunity and Cancer

### Rheumatoid Arthritis

Rheumatoid arthritis (RA) is an autoimmune disease where immune destruction of the synovial joints results in inflammation and pain. Although RA is considered an autoimmune disease and candidate autoantigens have been identified, the etiology of RA remains unknown (Chauhan et al., 2021). A commonly used animal model of RA is the K/BxN mouse which express the KRN T cell receptor transgene and the MHC-II molecule IAg7 (Ditzel, 2004; Monach et al., 2007, 2008). Autoantibody-induced arthritis (AIA) can be induced by the transfer of serum from K/BxN mice to recipient mice. Mast cells are increased in the synovial compartments in RA patients and synovial fibroblasts in AIA produce IL-33, which activates mast cells to release inflammatory mediators including IL-17, IL-13, GM-CSF, MCP-1, and MIP-1α (Robbie-Ryan and Brown, 2002; Table 1). These molecules recruit inflammatory cells and promote an autoreactive disease phenotype (Hueber et al., 2010; Xu et al., 2010). Mast cell-deficient mice showed little to no joint inflammation after serum transfer and disease susceptibility was restored after reconstitution of mast cells (Lee et al., 2002; Robbie-Ryan and Brown, 2002; Walker et al., 2012). These observations identify mast cells as a potential important link between autoantibodies and inflammatory arthritis.

Other forms of arthritis, such as osteoarthritis, have also been associated with mast cells. Studies have found mast cells and their mediators in the synovial and synovium fluids of individuals with osteoarthritis (Wang et al., 2019). Additionally, it was also discovered that IgE-mediated mast cell activation as well as mast cell-derived tryptase play a pathogenic role in the development of osteoarthritis (Wang et al., 2019). It is important to note that synovial mast cells in RA patients differ from those present in osteoarthritis patients in that the complement component 5a receptor 1 (C_5__*a*_) receptor is only expressed on RA synovial mast cells (Min et al., 2020). Mast cells have various methods of activation which include Fc receptors. The main method of activation is IgE-FcεR interaction, however, mast cells also express the FcγR and can be activated via IgG-FcγR interaction. Importantly, IgG is the major immunoglobulin produced in chronic inflammatory conditions such as RA (Min et al., 2020). Human synovial mast cells express multiple Fc receptors including FcεRI, FcγRI and FcγRII and the release of PGD2 and TNF is mediated by FcγRI-IgG interaction (Lee et al., 2013). Autoantibodies, including anti-citrullinated protein antibody (ACPA), play a critical role in RA pathogenesis in seropositive RA patients (Malmström et al., 2017; Aletaha and Smolen, 2018; Smolen et al., 2018). In human RA synovium, mast cells activated via IL-33 and ACPA immune complexes increase IL-8 and TNF production as well as IL-10 and histamine secretion (Kashiwakura et al., 2013; Rivellese et al., 2015).

Rheumatoid arthritis is positively associated with both Hodgkin’s and non-Hodgkin’s lymphoma, skin cancer, and lung cancer (Mellemkjaer et al., 1996; Askling et al., 2005). RA patients have a nearly two-fold increased risk of developing lymphoma compared to the general population (Klein et al., 2018). Patients with severe longstanding RA have a 70-fold increased risk of developing lymphoma compared to an 8-fold increase in those with intermediate disease. This suggests that risk is dependent upon RA disease severity. These findings further emphasize the link between inflammation and cancer. Treatment for RA involves the use of immunosuppressive therapies including anti-TNF blockers or tumor necrosis factor inhibitors (TNFi; Klein et al., 2018). It is therefore difficult to discern with certainty whether the increase in cancer incident in RA patients is a result of increased immune activation from the disease or a decrease in proinflammatory and antitumor immune responses from the treatments. RA patients given TNF antagonists had cancer incidences similar to other RA patients suggesting that immunosuppression may not be responsible for the cancer incidence increase and possibly pointing to severity of immune activation as the greater risk factor (Askling et al., 2005; Table 2).

**TABLE 2:** Autoimmunity, mast cell association, and associated cancers.

Autoimmune disease	Mast cell association	Associated cancer	References
Rheumatoid arthritis	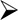 Mast cell mediators promote immune cell recruitment and angiogenesis 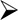 Mast cells increased in synovial compartments	Skin, lung, Hodgkin’s and non-Hodgkin’s lymphoma	Mellemkjaer et al., 1996; Lee et al., 2002, 2013; Robbie-Ryan and Brown, 2002; Askling et al., 2005; Hueber et al., 2010; Xu et al., 2010; Walker et al., 2012; Kashiwakura et al., 2013; Rivellese et al., 2015; Malmström et al., 2017; Aletaha and Smolen, 2018; Klein et al., 2018; Smolen et al., 2018; Min et al., 2020
Multiple sclerosis	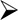 Mast cell mediators promote vascular permeability and immune cell recruitment 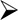 Mast cells present at sites of inflammatory demyelination 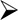 Mast cell density is increased in MS plaques	Respiratory, urinary, breast, CNS, non-melanoma skin cancer	Dietsch and Hinrichs, 1989; Galli et al., 1992; Brenner et al., 1994; Rozniecki et al., 1995; Ibrahim et al., 1996; Dimitriadou et al., 2000; Secor et al., 2000; Brown et al., 2002; Lock et al., 2002; Rottem and Mekori, 2005; Couturier et al., 2008; Sayed et al., 2008; Kingwell et al., 2012; Karagkouni et al., 2013; Sun et al., 2014; Grytten et al., 2020; Magyari and Sorensen, 2020
Type 1 diabetes	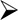 Increase in pancreatic mast cell density that correlates with apoptotic beta cells	Stomach, lung, liver, pancreas, kidney, endometrium, ovarian	Esposito et al., 2001; Geoffrey et al., 2006; Vigneri et al., 2009; Martino et al., 2015; Solt and Burris, 2015; Betto et al., 2017; Sona et al., 2018; Suh and Kim, 2019
Inflammatory bowel disease	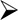 Increased mast cell density in ileum 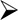 Increased histamine and histamine metabolites	Colorectal cancer, small bowel adenocarcinoma, intestinal lymphoma, anal cancer, cholangiocarcinoma	Lloyd et al., 1975; Fox et al., 1990; Nolte et al., 1990; Braegger et al., 1992; Traynor et al., 1993; Crowe et al., 1997; Gelbmann et al., 1999; Lilja et al., 2000; Oprins et al., 2000; Weidenhiller et al., 2000; Sasaki et al., 2002; Winterkamp et al., 2002; He, 2004; Jacob et al., 2005; Strober et al., 2007; Bischoff, 2009; Kurashima et al., 2013; Axelrad et al., 2016

### Multiple Sclerosis

Multiple sclerosis (MS) is a demyelinating disease of the central nervous system (CNS) in which the myelin sheath that insulates nerve cells in the brain and spinal cord are damaged by autoreactive immune cells. It is characterized by the presence of inflammatory lesions and demyelinating plaques. During MS, disruption of the nervous system’s ability to transmit signals results in double vision, muscle weakness, memory loss, and sensory dysfunction (Chiaravalloti and DeLuca, 2008). A commonly used animal model of MS is the experimental autoimmune encephalomyelitis (EAE) mouse. The EAE mouse is a CD4^+^ T cell-mediated autoimmune disease model characterized by inflammation in the CNS. As in MS, EAE mice experience a breach of the blood–brain barrier (BBB) allowing for inflammatory infiltrate into the CNS and destruction of myelin and myelin-producing cells (Olitsky and Yager, 1949; Wekerle, 2008). Mast cells have been observed at sites of inflammatory demyelination in the brain and spinal cord of MS patients and mast cell density is increased in CNS plaques of MS patients and EAE mice (Ibrahim et al., 1996; Karagkouni et al., 2013). Additionally, the proportion of degranulated mast cells in the CNS correlates with the clinical onset of disease symptoms in EAE and elevated levels of tryptase are found in the cerebrospinal fluid of MS patients (Dietsch and Hinrichs, 1989; Brenner et al., 1994; Rozniecki et al., 1995; Secor et al., 2000). Activated mast cells can produce IL-17, favoring the differentiation of CD4^+^ T cells into the Th17 phenotype (Piconese et al., 2009; Hemdan et al., 2010; Zhu and Paul, 2010). In the brains of MS patients, gene microarray data found elevated transcript levels of IL-17, real time PCR analysis revealed increased expression of CCL5 and SCF (two potent mast cell chemoattractants), and of mast cell-associated genes tryptase, chymase, and FcεRI β chain (Lock et al., 2002; Couturier et al., 2008). Inhibition of mast cell degranulation inhibits EAE onset and mast cell deficient mice have delayed disease onset and lower EAE disease scores when compared to controls (Dietsch and Hinrichs, 1989; Dimitriadou et al., 2000; Secor et al., 2000; Brown et al., 2002). Reconstitution of mast cell deficient mice with immature mast cells restores EAE susceptibility (Galli et al., 1992; Brown et al., 2002; Sayed et al., 2008). These findings suggest that mast cells may play an important role in MS development (Brown et al., 2002; Rottem and Mekori, 2005).

The release of histamine from mast cells promotes vascular permeability and allows for an influx of immune cells, including neutrophils, to the site of inflammation. Importantly, recent studies have demonstrated that neutrophils are required for BBB permeability and MS disease onset (Brown et al., 2002; Carlson et al., 2008). Therefore mast cells may have an indirect role in the development of MS via the recruitment of neutrophils to facilitate BBB permeability and initiate disease. A Norwegian study published in 2019 compared the risk of cancer among individuals with MS, their siblings, and the general population. It was reported that MS patients have an increased risk of respiratory (66% increase), urinary (51% increase), and CNS (52% increase) cancer development compared to the population controls. It was also reported that Taiwanese MS patients have a higher risk of developing breast cancer compared to controls (Sun et al., 2014). The overall cancer risk for people with MS was 14% higher than for individuals without MS (Grytten et al., 2020). However, another study reported an overall decrease in cancer incidence among MS patients with significant decreases in colorectal and stomach cancers and an increased risk of non-melanoma skin cancer (Kingwell et al., 2012). The discrepancy in MS-associated cancer risk may be due to varying disease severity or the patients susceptibility to comorbidities (Magyari and Sorensen, 2020; Table 2).

### Type 1 Diabetes

Type 1 diabetes (T1D) results from an autoimmune attack against the insulin-producing β-cells in the islets of Langerhans of the pancreas. The resulting failure of glucose homeostasis damages blood vessels and nerves (Bluestone et al., 2010). A widely used mouse model to study T1D is the non-obese spontaneously diabetic mouse (NOD). NOD mice exhibit immune dysregulation, like that observed in human T1D, resulting in the expansion of autoreactive T cells, autoantibody production, as well as the activation of innate immune cells that destroy insulin-producing β-cells. In the healthy pancreas, very few mast cells are present; however, in individuals with T1D, an increase in pancreatic mast cell density is positively correlated with the proportion of apoptotic β-cells (Esposito et al., 2001; Martino et al., 2015). Selective depletion of mast cells in NOD mice delays disease progression and treatment of NOD mice with the mast cell stabilizer cromolyn was also successful at delaying disease onset (Geoffrey et al., 2006; Betto et al., 2017). The release of IL-6 by activated mast cells in the pancreas favors a pathogenic Th17 phenotype associated with T1D (Solt and Burris, 2015; Betto et al., 2017). Together these findings suggest that mast cells and their inflammatory mediators have an important role in T1D disease development.

Individuals with diabetes mellitus (DM) have a 20–25% increased risk of developing cancer compared to those without DM (Vigneri et al., 2009). Specifically, individuals with T1D have been reported to have increased incidence of stomach, lung, liver, pancreas, kidney, endometrium, and ovarian cancers. T1D is also associated with an increased overall risk of cancer incidence and mortality (Sona et al., 2018; Suh and Kim, 2019). However, the mechanisms underpinning the association between T1D, and cancer remain unclear. Potential biological mechanisms may include insulin resistance that alters the risk of cancer (Giovannucci et al., 2010; Gordon-Dseagu et al., 2013). Additionally, T1D generally requires lifelong delivery of exogenous insulin. The insulin excess and possible mutagenic effects of insulin or insulin analog may also contribute to the increased risk of cancer development (Sona et al., 2018; Table 2).

### Inflammatory Bowel Disease

Inflammatory bowel disease (IBD), is a term encompassing two conditions, Crohn’s disease (CD) and ulcerative colitis (UC). IBD is characterized by chronic inflammation of the gastrointestinal tract caused by an abnormal immune response to intestinal flora. CD can affect any part of the gastrointestinal tract and most often affects the terminal ileus of the small intestine before the large intestine/colon while UC is mainly restricted to the large intestine and rectum (Fakhoury et al., 2014). Chronic inflammation of the gastrointestinal tract can often result in tissue damage.

Intestinal mast cells aid in the preservation of gut homeostasis via the maintenance of intestinal epithelium architecture and clearance of pathogens (Kurashima et al., 2013). Murine mucosal mast cells express MCPT-1 and MCPT-2 and produce lower levels of histamine compared to connective mast cells located in the submucosa (Bowcutt et al., 2014). Similar heterogeneity is observed in the human gastrointestinal tract, approximately 2–3% of total mucosal mast cells are MC_*T*_ and approximately 1% are of the MC_*TC*_ subtype populating the submucosa (Schemann and Camilleri, 2013). Effector functions also differ between mast cells of the small and large intestine. Bacterial colonization of the small intestine is lower than that of the colon (Shea-Donohue et al., 2010). Due to the lower bacterial burden in the small intestine, mast cells present in this region have lower expression of TLRs than those residing in the colon (Shea-Donohue et al., 2010). The dynamic microenvironment of the intestinal tract suggests that the phenotype of resident mast cells depends highly on the complex combination of factors present in the target tissue (Hamilton et al., 2014).

Increased mast cell density has been found in the ileum of patients with CD and UC (Nolte et al., 1990; Bischoff, 2009). Importantly, contents of the intestinal mast cells differed between IBD subjects compared to controls. TNF-positive mast cells were increased in the muscularis propria of patients with CD and increased levels of TNF was also detected in feces of patients with IBD (Braegger et al., 1992; Lilja et al., 2000). Laminin, a non-collagenous glycoprotein found in the extracellular matrix, was present in mast cells in the muscularis propria but not in those in the submucosa. These findings suggest that mast cells may be involved in remodeling of inflamed tissue (Gelbmann et al., 1999; Sasaki et al., 2002; He, 2004). Evidence of mast cell degranulation has also been observed in CD (Lloyd et al., 1975). Histamine secretion is increased in CD patients and N-methylhistamine, a metabolite of histamine, is increased in the urine of patients with active CD and UC (Weidenhiller et al., 2000; Winterkamp et al., 2002). Mast cells associated with active UC also release high levels of PGD2 (Fox et al., 1990). Diarrhea is a common occurrence in IBD cases and mast cell-derived tryptase can facilitate the disruption of epithelial junctions. Both histamine and TNF can increase ion transport across the epithelial barrier resulting in enhanced epithelial permeability and fluid transport (Traynor et al., 1993; Crowe et al., 1997; Oprins et al., 2000; Jacob et al., 2005; Strober et al., 2007). As a result of chronic intestinal inflammation, individuals with IBD are at an increased risk of developing intestinal malignancies including colorectal cancer (CRC), small bowel adenocarcinoma, intestinal lymphoma, anal cancer, and cholangiocarcinoma (Axelrad et al., 2016; Table 2).

### Mast Cells and Cancer Development

Tumor cells produce several chemotactic factors, such as SCF, VEGFs, and CXCL8/IL-8, that attract mast cells to the tumor microenvironment (TME; Varricchi et al., 2017). The TME is composed of various other cell types including stromal cells, neutrophils, macrophages, and T and B cells. Once in the TME, mast cells mature and begin to produce angiogenic mediators and growth factors that promote tumor growth (Huang et al., 2008; Vahidian et al., 2019). Tumor-associated mast cells (TAMC) help to regulate angiogenesis and are attributed to both tumor promotion and tumor rejection (Aoki et al., 2003; Beer et al., 2008; Johansson et al., 2010; Rabenhorst et al., 2012; Ma et al., 2013; Varricchi et al., 2017). Angiogenesis supplies growing tissue with vital nutrients like oxygen and is essential for tumor growth, invasiveness, and metastasis (Derakhshani et al., 2019). Production of CXCL12 by tumor cells attracts mast cells to the TME and promotes secretion of pro-angiogenic factors such as VEGF-A, heparin, histamine, and SCF (Jorpes, 1959; Theoharides et al., 1982; Kim et al., 1993; Kryczek et al., 2005; Beer et al., 2008; Carmeliet and Jain, 2011; Kolset and Pejler, 2011; Vizio et al., 2013; Derakhshani et al., 2019). Additionally, mast cells promote tumor progression by suppressing immune responses via the release anti-inflammatory cytokines, such as IL-10 and TGFβ (Riley and West, 1952; Nakae et al., 2005; Melillo et al., 2010; Ribatti et al., 2010; da Silva et al., 2014; Krystel-Whittemore et al., 2015; Derakhshani et al., 2019). Tregs, T effector cells, and mast cells often co-localize at sites of inflammation and specific tumor microenvironments. Tregs have a crucial role in regulating immune responses, however, mast cells can reverse Treg suppression of T effector cells (Gri et al., 2008; Kashyap et al., 2008; Piconese et al., 2009). Tumor cells and mast cells also produce immunosuppressive adenosine which is increased in the TME. Adenosine promotes histamine secretion from mast cells which stimulates vasodilation and immune suppression by inhibiting Th1 and Th2 responses through activation of H2 receptors (Riley and West, 1952; Marquardt et al., 1984; Gottfried et al., 2012; Piliponsky et al., 2012). Mast cells also produce CXCL8/IL-8 which can induce epithelial-to-mesenchymal transition (EMT) in which tumor cells acquire metastatic features and resistance to chemotherapy (Scheel and Weinberg, 2012; Visciano et al., 2015; Zheng et al., 2015). Localization and differences in mast cell density between the periphery and center of tumors is also important in the prognosis of specific cancers. In pancreatic adenocarcinoma, mast cell density in the intratumoral border region is associated with a worse prognosis. Conversely, high intratumor mast cell density in human prostate cancer is associated with inhibition of tumor growth and a better prognosis (Fleischmann et al., 2009; Johansson et al., 2010; Cai et al., 2011). The alarmin IL-33 is upregulated in squamous cell carcinoma and activates mast cells by binding to its surface receptor, ST2. Activation of mast cells via IL-33 promotes the production and release of IL-13, CXCL8/IL-8, and VEGF-A from mast cells, further influencing the inflammatory response (Theoharides et al., 2010; Byrne et al., 2011; Chan et al., 2012; Rivellese et al., 2015; He et al., 2018; Table 1).

Chronic inflammation increases an individual’s risk of developing precancerous lesions and adenocarcinomas. The inflamed regions surrounding gastric ulcers increase an individual’s risk of developing gastric cancer and IBD increases one’s risk of developing CRC. In cases of CRC, studies have implicated the innate immune system in the development of precancerous adenomatous polyps (Horton et al., 2000; Itzkowitz and Yio, 2004; Triantafillidis et al., 2009; Terzić et al., 2010). In gastric cancer cases, mast cell density is increased in well-differentiated gastric tumors and likely the source of pro-angiogenic and pro-tumorigenic factors (Mukherjee et al., 2009). Additionally, mast cell density is correlated with the degree of gastric tumor vascularization as stage IV gastric tumors have increased mast cell density and vascularization compared to early stage tumors (Ribatti et al., 2010). Similar to the findings in gastric cancer patients, a correlation was also found between the degree of angiogenesis and mast cell density in CRC. Patients with tumors of low vascularization and low mast cell density had longer survival rates than those with high degrees of vascularization and high mast cell density. High TAMC density may be a useful biomarker for predicting poor survival of patients with CRC (Yodavudh et al., 2008; Hodges et al., 2012). Mast cells are pro-tumorigenic in several solid tumors, such as gastric, pancreas, and thyroid, yet they have also been found to have a protective anti-tumor role in intestinal tumorigenesis. Epidemiologic evidence has suggested a negative association between the presence of mast cells and tumor progression in lung and breast cancers (Sinnamon et al., 2008; Hodges et al., 2012). Together these findings show that the role of mast cells, whether pro-tumorigenic or anti-tumorigenic, is cancer specific and depends greatly on the location and phenotype of the infiltrating mast cells.

## Mast Cells as a Potential Therapeutic Target

Several different methods of targeting and controlling mast cells and mast cell activation have been developed as potential therapeutics (Figure 1). Sialic acid-binding immunoglobulin-like lectins (Siglec)-8 is expressed on the surface of human eosinophils, mast cells, and basophils. Antibody-mediated engagement of Siglec-8 (lirentelimab) inhibits mast cell degranulation. A humanized IgG1 antibody against Siglec-8 is in clinical development for the treatment of mast cell-mediated diseases (Kiwamoto et al., 2012; O’Sullivan et al., 2018; Youngblood et al., 2020). Molecules like programmed cell death-1 (PD-1) ligands (PD-L1 and PD-L2) are upregulated on tumor cells, impeding the activation of PD-1^+^ effector cells upon ligand-receptor interaction (Sharma and Allison James, 2015). Human mast cells express PD-L1 and PD-L2. There are monoclonal antibodies targeting the PD-1/PD-L1 pathway (nivolumab, pembrolizumab, avelumab, atezolizumab, and durvalumab) that provide antitumor immunity by removing this impediment and may be a potential therapeutic method for inhibiting mast cell-associated tumor growth (Varricchi et al., 2017; Lv et al., 2019; Zhao et al., 2020). Mast cells promote proliferation of colon cancer cells and depletion of mast cells via injection with the chimeric toxin Fce-PE40, which binds to mouse FcεR, was shown to decrease colon tumor weight and volume without inducing mast cell degranulation or anaphylaxis *in vivo* (Wang et al., 2016). Symptoms of mast cell activation or mediator release are treated with H_1_/H_2_ antihistamines and mast cell stabilizers (Lee and Akin, 2013). Continuous infusion of diphenhydramine, a histamine H_1_ receptor blocker, has been used to suppress mast cell activation. Additionally, glucocorticoids aid in the reduction of SCF and mast cell activation. Omalizumab, a humanized anti-IgE murine monoclonal antibody, binds to the domain of the Fc region of IgE preventing mast cell activation and has been deemed safe and effective in case reports of mast cell activation disease (MCAD; Molderings et al., 2016). TNF is critical in the pathogenesis of several inflammatory diseases. Anti-TNF antibodies (infliximab, adalimumab) have become important for the treatment of CD and RA (Adegbola et al., 2018). Sulfasalazine has been used to treat IBD and is also shown to inhibit MC mediator secretion (Bissonnette et al., 1996). It is likely that mast cells play a role in cancer development or progression via regulation of inflammatory responses. These aforementioned approaches may be effective therapeutic strategies for preventing or treating various autoimmune diseases and cancers.

## Conclusion

Mast cells have a high degree of heterogeneity, and the inflammatory response of the surrounding immune cells have considerable influence on mast cell phenotype. Conversely, mast cells can influence the immune response in some cancers and tissues affected by autoimmunity. It is therefore important to identify the stimuli that can activate mast cells in different disease settings and tumor microenvironments. Additionally, mast cells within inflamed tissues and tumors may have either pro- or anti-tumorigenic properties. Questions remain regarding how mast cells are activated, the extent of mast cell involvement in various diseases, and the role of mast cells in early- versus late-stage tumors. Though these questions remain unanswered, the disease-exacerbating role for mast cells in autoimmunity and the development of certain cancers cannot be ignored. This review summarized findings that implicate mast cells as a potential therapeutic target to possibly alter disease onset, severity, and progression in patients.

## Author Contributions

CN took the lead on conceptualization of the review topic and gathered information referenced in the manuscript. SH assisted in manuscript revision. RD supported in manuscript revision and organization. All authors contributed to the article and approved the submitted version.

## Conflict of Interest

The authors declare that the research was conducted in the absence of any commercial or financial relationships that could be construed as a potential conflict of interest.

## Publisher’s Note

All claims expressed in this article are solely those of the authors and do not necessarily represent those of their affiliated organizations, or those of the publisher, the editors and the reviewers. Any product that may be evaluated in this article, or claim that may be made by its manufacturer, is not guaranteed or endorsed by the publisher.
